# A Unified, One Fluid Model for the Drag of Fluid and Solid Dispersals by Permeate Flux towards a Membrane Surface

**DOI:** 10.3390/membranes11020154

**Published:** 2021-02-22

**Authors:** Amgad Salama, Shuyu Sun, Tao Zhang

**Affiliations:** 1 Process System Engineering, University of Regina, Regina, SK S4S 0A2, Canada; 2 Physical Science and Engineering Division, King Abdullah University of Science and Technology, Thuwal 23955, Saudi Arabia; shuyu.sun@kaust.edu.sa (S.S.); tao.zhang.1@kaust.edu.sa (T.Z.)

**Keywords:** drag on a droplet, oily water filtration, membrane technology, one fluid model, finite volume method

## Abstract

The drag of dispersals towards a membrane surface is a consequence of the filtration process. It also represents the first step towards the development of the problem of fouling. In order to combat membrane fouling, it is important to understand such drag mechanisms and provide a modeling framework. In this work, a new modeling and numerical approach is introduced that is based on a one-domain model in which both the dispersals and the surrounding fluid are dealt with as a fluid with heterogeneous property fields. Furthermore, because of the fact that the geometry of the object assumes axial symmetry and the configuration remains fixed, the location of the interface may be calculated using geometrical relationships. This alleviates the need to define an indicator function and solve a hyperbolic equation to update the configuration. Furthermore, this approach simplifies the calculations and significantly reduces the computational burden required otherwise if one incorporates a hyperbolic equation to track the interface. To simplify the calculations, we consider the motion of an extended cylindrical object. This allows a reduction in the dimensions of the problem to two, thereby reducing the computational burden without a loss of generality. Furthermore, for this particular case there exists an approximate analytical solution that accounts for the effects of the confining boundaries that usually exist in real systems. We use such a setup to provide the benchmarking of the different averaging techniques for the calculations of properties at the cell faces and center, particularly in the cells involving the interface.

## 1. Introduction

The problem of fouling is considered the most serious problem that can hinder the operational effectiveness of membranes and considerably shorten their lifetime. Therefore, there are a large amount of research works that have been devoted to investigate this phenomenon, highlight the factors that may affect its development, and propose methodologies to combat its progression [1,2]. Fouling is the outcome of two phenomena—namely, (1) the selectivity feature of the membrane and (2) the hydrodynamic drag that dispersals encounter towards the membrane surface [2,3,4,5,6,7,8], to list but a few. The selectivity feature of membranes is essentially related to the sizes of their pores in relation to the sizes of dispersals. Accordingly, if the largest size membrane pores are smaller than the smallest size dispersals, no permeation of dispersals would occur and a complete pure filtrate would be obtained. On the other hand, when the dispersals represent droplets of immiscible fluids, it becomes unclear how the selectivity of the membrane would work. Fluids can easily squeeze, deform, and take the shape of the openings, no matter what sizes they are. However, when surface forces are utilized, interesting outcomes may be obtained. If the surface of the membrane is chosen to be nonwetting to the dispersed droplets, interfaces are formed at the pore openings of the membrane when droplets land over the surface. Such interfaces prevent droplets from permeation if the pressure in the feed channel is kept smaller than a threshold value called the critical entry pressure. Since pores and droplets assume distributions of sizes, then multiple such critical threshold pressures exist. This implies that there would always be droplets forming over pore openings where the applied pressure is larger than the critical pressure and, therefore, permeation occurs and vice versa.

Particles and/or droplets are dragged towards the membranes’ surface by the virtue of permeation flux. Therefore, it is essentially hydrodynamic drag that brings the dispersal towards the surface of the membrane. Furthermore, in crossflow filtration hydrodynamic drag sweeps off and detaches pinned droplets. The study of the drag forces over immersed solid or fluid particles is fundamentally important in order to develop methods to reduce the accumulation of dispersals over the surface of the membrane. Accordingly, there is a long pathway of continuous research to reach a suitable framework to handle this interesting phenomenon. Although there exists number of algorithms, the most widely used technique that can handle a collection of particles is within the discrete element methods in which the Navier–Stokes equations are solved in the background and the drag over the particles and the interactions between particles are modeled [8]. While this can be useful, the accurate representation of drag force over immersed objects remains challenging. In particular, for the case of droplets the drag force is apparently dependent on the viscosity contrast between the continuous phase and the dispersed droplets [9]. In other words, even at the scale of a single droplet, there are still issues related to the correct representation of the drag force over a wider range of Reynolds numbers along with different viscosity contrasts. The traditional methods for handling this problem involve a sharp interface and diffuse interface models that are very expensive computationally and require exhaustively denser meshes [2,3,10,11,12,13,14]. In this work, we introduce an algorithm that may work not only to study the drag over solid particles but also that over droplets. This methodology does not require a denser mesh and therefore is computationally very effective. However, it will only apply to scenarios in which the interface maintains its configuration. This would be the case for solid particles or droplets of spherical or cylindrical shapes. Furthermore, it can also handle solid particles that are relatively large in size. Numerically, both solid particles and droplets are handled differently. In the case of a spherical solid particle moving in a quiescent fluid, the fluid domain, excluding the particle, is discretized and the solution is obtained within the fluid domain [15,16,17,18,19,20]. This will not be the case when the spherical object is a droplet of an immiscible fluid [9,10,11,12,13,14]. In this case, the whole domain, including the droplet, should be discretized to correctly capture the velocity and pressure fields.

The moving fluid around suspended droplets generates surface forces per unit area, the integration of which over the area in the direction of the flow defines the forces of interaction between the external fluid and the suspended droplets. When the suspended objects are rigid, it may be relatively easy to analyze such interactions for relatively simple geometrical shapes [21,22]. When the objects are other fluids immiscible with the continuous one, the interface boundaries between phases assume relatively complex and dynamic configurations, which make it challenging to analyze their evolution. Furthermore, it becomes important to analyze the surface forces at both sides of the interface in order to link the flow fields between the different phases. The treatment of interfacial coupling between phases gives rise to a possible jump in normal stresses across the interface. The interesting thing, however, is related to whether there also exists a jump in the shear stress at the interface or not [23,24,25]. This has been a topic of an old debate when Rybczynsk [23] and Hadamard [24] independently developed an analytical solution to the problem of estimating the drag force over a spherical droplet in the creep flow regime. In their derivation, they assumed no jump in the shear stress at the interface. Boussinesq [25] later developed a constitutive relationship to account for the effect of the interface in which he introduced two types of surface viscosities.

**Figure 1:**
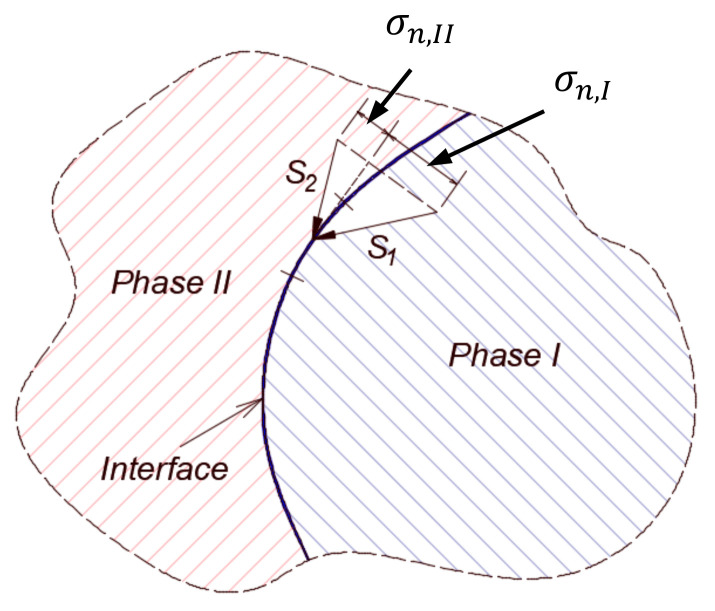
Surface forces at the two sides of the interface between two phases. Jump in the normal stress is correlated with the curvature of the interface. Tangential stress does not experience jump under the no-slip condition.

In the context of this work, we adopt the assumption of no jump in the shear stress on the interface. Our motive in this stems from the sharp interface limit in which the interface represents a surface of discontinuity deprived from any physical properties [26,27]. Such an assumption, together with the sharp interface limit argument, implies the following two points—namely, (1) no jump in the velocity at the interface, (2) no jump in the shear stress along the interface as long as there is no gradient in the interfacial tension. The continuity of the normal component of velocity complies with the mass conservation law, while the continuity of the tangential components of velocity complies with the regular no-slip condition. In a future work, we shall consider the cases when these conditions are not satisfied. The jump in normal stress exists in both scenarios and is responsible for the curvature of the interface between two immiscible fluids; see Figure 1. The treatment of the motion of a multiphase system can be quite complex. In general, the obvious, but not necessarily the easiest method is to consider a different set of equations for each fluid phase. In the context of the general flow field (i.e., the overall domain), this requires the coupling of the different fluid domains together through conditions at the interface, which would essentially require the continuous monitoring and tracking of the interfaces. This can be computationally expensive, particularly if there exist several such interfaces. Other techniques exist that are customized to different situations in order to reduce the computational burden, which will be briefly discussed in the next section.

The aim of this work is not, therefore, to model fouling development. Rather, it is to model the drag of dispersals towards the membrane by virtue of permeation flux. Even though such fluxes are usually small, they are able to bring dispersals to the membrane. The aim of this work is, therefore, to provide an algorithm that would be able to handle this system with less computational burden compared with other methods. A framework is introduced in which both solid particles and droplets are dealt with in a unified way. This framework assumes both the solid particles and droplets as fluids with a sharp contrast in both the density and viscosity fields. Since we are particularly interested in small-scale dispersals (of sizes in the order of tens of microns), droplets are assumed to maintain their spherical shape. In other words, surface forces are assumed to not play a significant role in the movement of droplets, and this significantly reduces the number of degrees of freedom associated with this system and, likewise, the runtime. It is to be mentioned that, in this part, we try to benchmark the capabilities of the developed algorithm and highlight the different techniques to handle the interfacial quantities. Since ample data for the drag of solid particles are available, the problem of settling of a solid cylindrical object is used as a benchmark case to provide a comparison and verification exercise.

## 2. Governing Equations

The drag force over an immersed solid object is the net surface force over the object in the direction of the flow. Therefore, if **S** represents the surface force per unit area due to the motion of the fluid relative to the object, then the net surface force in the direction of the bulk motion may be expressed as:

(1) $$F_{D}=\underset{A}{\int }S\cdot edA,$$

where $F_{D}$ is the drag force and **e** is the unit vector in the direction of the bulk flow field. The surface force per unit area, **S**, is correlated with the stress tensor at the surface, such that S=Tn, where T is the stress tensor and **n** is the outwardly unit normal vector to the area. The stress tensor for incompressible Newtonian fluids can be written as T=−pI+τ, where $\tau =\mu \left(\nabla v+\nabla v^{T}\right)$ is the viscous stress tensor. This formulation suggests that there exist two contributions into the hydrodynamic drag—one contribution from the pressure field (this is called form drag) and another contribution from viscous force (this is called skin drag). In order to accurately determine the drag force, both the velocity and the pressure fields around the object need to be resolved.

The problem will even be more complicated should the object be another fluid immiscible with the surrounding fluid. The governing equations that are applicable to all phases represent conservation laws of mass and momentum [26,27,28,29,30,31,32]. As mentioned, these equations can apply to all fluid regions augmented with equations at the interface between phases. Such an approach may be known as a multidomain approach, in which each domain is described by different fluid properties that are generally homogeneous (in cases where no heat or species transport occur). Therefore, $\Omega \in R^{3}$ is the computational domain, which contains a two-phase system. We are particularly interested in the case in which one of the phases is completely enclosed inside the other phase. For the time-dependent problem (within the time period [0, T]), the two domains are defined as $\Omega_{1}\left(t\right)$ and $\Omega_{2}\left(t\right)$, with $\bar{\Omega }={\bar{\Omega }}_{1}\cup {\bar{\Omega }}_{2}$ and $\Omega_{1}\cap \Omega_{2}=\emptyset$. In this case, the interface is defined as $\Gamma \left(t\right)={\bar{\Omega }}_{1}\left(t\right)\cap {\bar{\Omega }}_{2}\left(t\right)$. Over the domain Ω, the governing equations summarizing the conservation laws can be written as:

Mass conservation:

(2) $$\underset{CV}{\int }\frac{\partial \rho }{\partial t}dv+\underset{A}{\int }\rho v\cdot ndA=0,\;$$

(3) $$\frac{\partial \rho }{\partial t}+\nabla \cdot \left(\rho v\right)=0{\;\mathrm{in}\;\Omega }_{1}\;\&{\;\Omega }_{2}\times \;\left[0,T\right],$$

Momentum conversation:

(4) $$\frac{\partial \rho }{\partial t}+\nabla \cdot \left(\rho v\right)=0{\;\mathrm{in}\;\Omega }_{1}\;\&{\;\Omega }_{2}\times \;\left[0,T\right],\;$$

(5) $$T=-\left(p+\frac{2}{3}\mu \nabla \cdot v\right)I+\mu \left(\nabla v+\nabla v^{T}\right),\;$$

(6) $$\frac{\partial \rho v}{\partial t}+\nabla \cdot \rho vv=-\nabla p+\nabla \cdot \mu \nabla v+\rho g{\;\mathrm{in}\;\Omega }_{1}\;\&{\;\Omega }_{2}\times \left[0,T\right].\;$$

In the above equations, v is the velocity vector [L/T], n is the outwardly unit normal vector, ρ is the density [M/L^3^], μ is the viscosity [M/LT], T is the stress tensor [M/LT^2^], *p* is the pressure [M/LT^2^], and g is the gravity.

The problem, however, with this approach is related to the need to describe auxiliary equations along the interfaces (i.e., along $\Gamma \left(x,t\right)$) that couple the different domains, which can be fairly complex. Another approach that is currently widely used is the so-called one-domain approach, which considers the fluid domains (including the different phases) as one domain that contains a nonhomogeneous fluid. The nonhomogeneity of the fluid implies that the fluid properties change spatially within the domain. To account for interfacial forces and the jump in normal stresses experienced at the interface, a volumetric force is added to the momentum equation to represent interfacial tension. In this case, the momentum equation may be written as:

(7) $$\frac{\partial \rho v}{\partial t}+\nabla \cdot \rho vv=-\nabla p+\nabla \cdot \mu \nabla v+\rho g+F_{\sigma },\;$$

where $F_{\sigma }$ is the interfacial tension force per unit volume. In the above formulation, the surface tension force is transformed to a volume force spread over a few layers of cells. In fact, the interfacial tension force per unit area at a point on the interface is given by Olsson and Kreiss [33]:

(8) $$F_{s,\sigma }\left(x_{i}\right)=\sigma \kappa \left(x_{i}\right)n\left(x_{i}\right),\;$$

where the subscript *i* refers to the interface. The non-dimensional incompressible Navier–Stokes equations for an incompressible two-phase system with surface tension and gravity, as suggested by Olsson and Kreiss [33], can be written as:

(9) $$\frac{\partial v}{\partial t}+\nabla \cdot vv=-\nabla p+\frac{1}{Re}\nabla^{2}v+\frac{1}{Fr^{2}}e_{g}+\frac{1}{We}F_{\sigma },$$

where $Re=\rho_{ref}U_{ref}L_{ref}/\mu_{ref}$ is the Reynolds number, $Fr=U_{ref}/\sqrt{L_{ref}g}$ is the Froude number, and $We=\rho_{ref}U_{ref}^{2}L_{ref}/\sigma$ is the Weber number. The volume force can be written as:

(10) $$F_{\sigma }=F_{V,\sigma }\left(x\right)=\sigma \left(-\nabla \cdot \frac{\nabla \varphi }{\left|\nabla \varphi \right|}\right)\nabla \varphi .$$

This will result in the same total force as $F_{s,\sigma }\left(x_{i}\right)$, but spread over the finite interface width. This interfacial force depends on the curvature of the interface, which therefore needs to be determined. The determination of the curvature of the interface requires a dynamic reconstruction of the interface with time and location. In order to reconstruct the interface, a phase binary function $\varphi \left(x,t\right)$ is defined. This function is assumed equals one when the position vector x lies inside one phase and is zero in the other phase. The dynamic behavior of this function is obtained by solving an advection differential equation of the form:

(11) $$\frac{\partial \varphi }{\partial t}+v\cdot \nabla \varphi =0.$$

Through the substitution of Equation (9) into Equation (7) and rearrangement, one obtains:

(12) $$\frac{\partial \rho v}{\partial t}+\nabla \cdot \rho vv=-\nabla p+\nabla \cdot \mu \nabla v+\rho g-c\nabla \varphi ,\;$$

where $c\left(x,t\right)\;=\sigma \nabla \cdot \left(\nabla \varphi /\left|\nabla \varphi \right|\right)$. Through the integration of the above equation over the computational domain, one obtains:

(13) $$\frac{\partial \rho v}{\partial t}+\nabla \cdot \rho vv=-\nabla p+\nabla \cdot \mu \nabla v+\rho g-c\nabla \varphi ,\;$$

(14) $$\underset{V}{\int }\left(\frac{\partial \rho v}{\partial t}+\nabla \cdot \rho vv\right)dV=\underset{V}{\int }\left(-\nabla p+\nabla \cdot \mu \nabla v+\rho g\right)dV-\underset{V}{\int }c\nabla \varphi dV.\;$$

**Figure 2:**
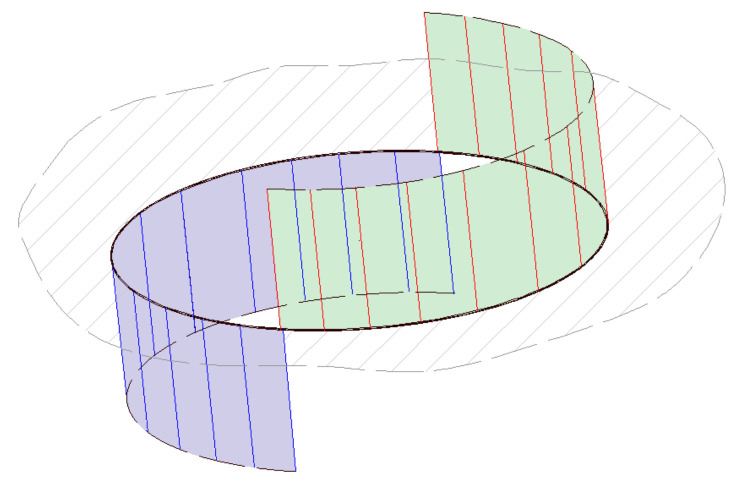
Schematic diagram of the gradient function of the phase function, φ. The gradient is zero in every way except at the interface, where it jumps as a positive delta function on half the interface and a negative delta function over the second half. Therefore, its integral over the whole interface is zero.

The last term can be written as: $\int_{V}\sigma \left[\nabla \cdot \left(\nabla \varphi /\left|\nabla \varphi \right|\right)\right]\;\nabla \varphi dV$. The curvature of the interface is defined as: $\kappa \left(x,t\right)=\nabla \cdot \left(\nabla \varphi /\left|\nabla \varphi \right|\right)$. The phase field function $\varphi \left(x,t\right)$ is zero in the continuous phase and one in the dispersed phase, and therefore ∇φ is zero in both the phases and shoots at the interface, as shown in Figure 2. In this case, the interfacial force term may be written as $\int_{V}\sigma \kappa \left(x,t\right)\nabla \varphi dV=\int_{V}\sigma \kappa \left(x,t\right)\delta \left(x_{I}\right)dV$, where $x_{I}$ is the position vector that marks the interfacial region. The previous formula may further be reduced to $\int_{V}\sigma \kappa \left(x,t\right)\delta \left(x_{I}\right)dV=$ $\int_{I}\sigma \kappa \left(x_{I},t\right)dV$, where the subscript *I* refers to the interfacial region. When the interface remains circular, $\delta \left(x_{I}\right)$ is positive along half the interface and negative in the second half, therefore $\int_{I}\sigma \kappa \left(x_{I},t\right)dV=\int_{I_{1}}\sigma \kappa \left(x_{I_{1}},t\right)dV-\int_{I_{2}}\sigma \kappa \left(x_{I_{2}},t\right)dV$. For the case where the interface maintains its circular or spherical shape, the curvature remains constant and the integral is zero. This leads the momentum equation to take the following form:

(15) $$\frac{\partial \rho v}{\partial t}+\nabla \cdot \rho vv=-\nabla p+\nabla \cdot \mu \nabla v+\rho g.$$

If the interfacial tension is not sufficiently strong and/or the size of the droplet is not sufficiently small, the droplet might deform during flow; then, the force term from the interfacial tension needs to be incorporated into the Navier–Stokes equation for the prediction of deformation.

However, as indicated earlier, in this work we are interested in objects that maintain their integrity and experience no deformation, which may be the case when the interfacial tension is large and/or the size of the droplet is small. In this case, the interfacial force is large enough to maintain the uniformity of the initial configuration (i.e., spherical in 3D and circular in 2D). In this case, the interfacial forces act to jump the pressure across the interface uniformly. In other words, they contribute uniformly to the distribution of the pressure field inside the droplet, which is a consequence of the induced flow due to the external drag. This implies that shifting the pressure field inside the droplet uniformly up or down (e.g., by adjusting the boundary conditions) does not influence the internal flow field. In other words, even though the pressure field inside the droplet may not be unique, the induced velocity field is unique. Thus, while the pressure field inside the droplet might miss an additive constant, this does not affect the pressure solution outside of the droplet and certainly does not change the velocity solution in the entire domain.

Furthermore, the fact that the droplet maintains its spherical shape makes it relatively easy to reconstruct the interface without the need to solve an extra equation for the phase indicator function, particularly as this equation is hyperbolic and could be prone to artificial diffusion. This significantly reduces the computational burden to fractions of those required when accounting for both interfacial forces and interface configuration. In several of our previous CFD investigations of the problem of permeation of a microscale oil droplet, a very small time step is required (in the order of 0.01 µs) to achieve the required tolerance with the total time of only fractions of a second [2,3,10,11,12,13,14]. In this present technique, the time step and spatial discretization can become considerably coarse compared with interface-resolving techniques. As has already been indicated, the purpose of this study is to only highlight the influence of both the external and internal flow fields on hydrodynamic drag on relatively simpler cases. For those particular cases involving complex interface configurations, the interface deformation becomes important, and therefore Equation (7) and the surface force term in Equation (6) would need to be accounted for.

As indicated, since in this work we will be concerned with mainly with cylindrical objects, in the next section we provide some of the analyses concerning the analytical difficulties associated with this system and the available approximate solution. These solutions will be used to benchmark this approach and provide confidence in the present numerical approach.

## 3. Framework for Validation

The validation exercise conducted in this work considers the terminal velocity when an object is set to move in a quiescent fluid according to Happel and Brenner [34]. The velocity of a particle settling in an infinite quiescent fluid increases until it reaches steady terminal value when the forces balance. Consider an isolated particle *i* being dropped in a fluid in the Stokesian creeping regime. Its motion is governed by:

(16) $$m_{i}{\dot{v}}_{i}=-c_{i}v_{i}-m_{i}g+\delta_{c},\;$$

(17) $${\dot{v}}_{i}=-\frac{9c_{0}}{2\rho_{i}R_{i}^{2}}v_{i}-g+\frac{\delta_{c}}{m_{i}},\;$$

(18) $$\alpha_{i}≝\frac{9c_{0}}{2\rho_{i}R_{i}^{2}},$$

where $\delta_{c}$ is a term to account for the collision of particles, $m_{i}=\rho_{i}\frac{4}{3}\pi R_{i}^{3},$ and the Stokesian drag is $c_{i}=c_{0}6\pi R_{i}$. Ignoring the collisions with other particles, the velocity can be determined to be:

(19) $$v_{i}\left(t\right)=v_{i}\left(t=0\right)e^{-\alpha_{i}t}+\frac{g}{\alpha_{i}}\left(e^{-\alpha_{i}t}-1\right).\;$$

The position is updated as:

(20) $$r_{i}\left(t\right)=r_{i}\left(t=0\right)+\frac{v_{i}\left(0\right)}{\alpha_{i}}\left(1-e^{-\alpha_{i}t}\right)+\frac{g}{\alpha_{i}}\left(\frac{1}{\alpha_{i}}-\frac{e^{-\alpha_{i}t}}{\alpha_{i}}+t\right).\;$$

The terminal velocity, therefore, is:

(21) $$v=\frac{g}{\alpha_{i}}.\;$$

In this work, the numerical scheme and the modeling framework is validated against the benchmark problem of a 2D cylinder settling between two vertical plates. This is due to the computational burden associated with the numerical solution of the three-dimensional problem regarding the settling of a spherical object in a quiescent fluid. It is interesting, however, to note that for two-dimensional streaming motion perpendicular to the axis of a circular cylinder, there exists no solution of the creeping motion equations, which is in contrast to the three-dimensional problem of flow past a sphere. Based on dimensional arguments, it is found that the force per unit length (*F*) on the body due to the flow scaled by the viscosity of the fluid and the velocity (i.e., F/μU) is the only dimensionless number that can be constructed and would need to be an equal constant. This apparently may not be possible, as it indicates that the force per unit length is independent of the size of the cylinder.

**Figure 3:**
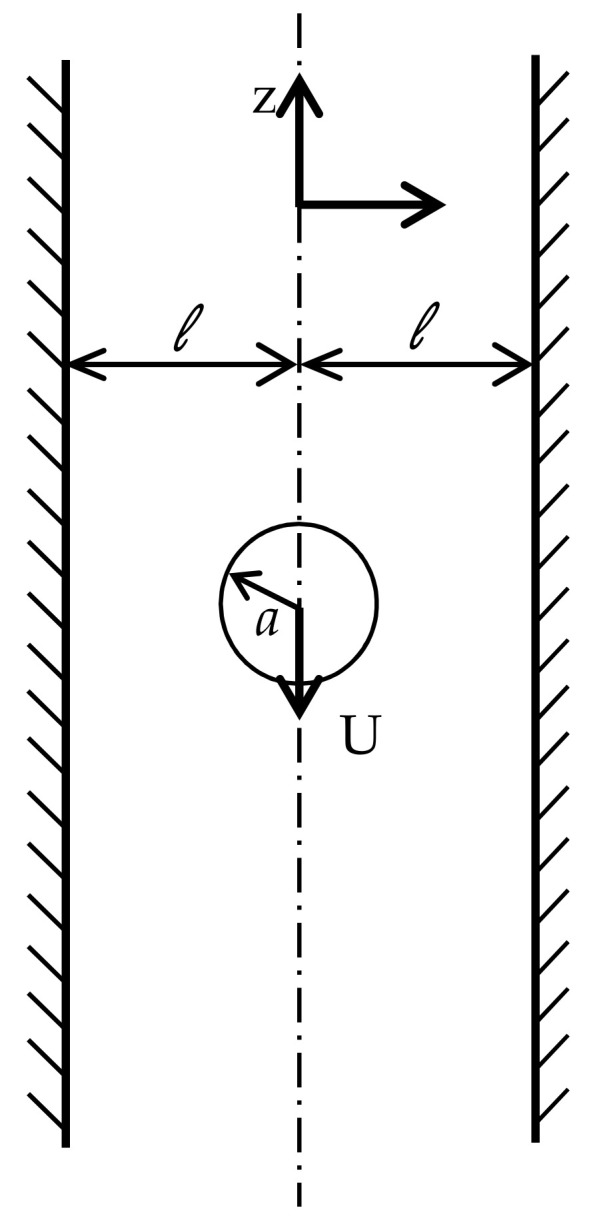
Schematic of the domain where Faxén [39] expansion apply.

Oseen [35] pointed out that the validity of Stokes’ equations relies on the Reynolds number being small, which cannot hold for arbitrarily large distances [36]. He suggested including inertial contributions to the momentum equations, and this enabled Lamb [37] to provide an approximate solution for cylinder drag in an infinite domain. The numerical solution of this problem in such an unbounded domain is clearly not possible. It has been determined that a domain size of up to 1150 radii may be needed, such that wall effects may be negligible [38]. This (among others) motivated Faxén [39] to search for an expansion that allows one to correct for possible boundary effects [39]. He was able to provide an approximate solution to predict the drag force on infinite cylinder moving between two parallel plates. The terminal settling velocity according to the Faxén expansion, Pianet and Arquis [40], is given as:

(22) $$U_{c}=\left(1-\frac{\rho_{p}}{\rho_{f}}\right)\frac{\rho_{f}ga^{2}}{4\mu_{f}}f\left(k_{x}\right),$$

(23) $$f\left(k_{x}\right)=-0.9157-\ln\left(k_{x}\right)+1.7244k_{x}^{2}-1.7302k_{x}^{4},\;$$

where $\rho_{p}$ is the density of the particle, $\rho_{f}$ is the density of the fluid, g is the gravity, *a* is the radius of the sphere, and $\mu_{f}$ is the viscosity of the fluid. The function $f\left(k_{x}\right)$ accounts for the wall correction with $k_{x}=a/l$ where l is half the width of the channel, as shown in Figure 3.

Several other researchers performed numerical investigations of the Faxén problem and verified his expansion [41,42]. As pointed out earlier, Pianet and Arquis [40] provided a benchmark analysis for validating the interaction of particle, fluid, and confining walls. They used a one-fluid model, in which both fluid and solid particles are considered as a fluid which requires equivalent densities and viscosities. In this framework, a phase function, $F\left(x,t\right),$ is introduced such that it is zero in the fluid region and one in the solid region. The fictitious fluid viscosity and density, $\mu_{l}$ and $\rho_{l}$ are therefore defined as $\mu_{l}=\mu_{f}\left(1-F\right)+\mu_{p}F$ and $\rho_{l}=\rho_{f}\left(1-F\right)+\rho_{p}F,$ where the subscripts *f*, *p*, and l denote the fluid, the solid particles, and the fictitious overall system, respectively. In this case, an additional equation for the convection of F is introduced, which takes the form:

(24) $$\frac{\partial F}{\partial t}+v\cdot \nabla F=0.$$

The numerical solution of this equation is challenging and prone to artificial numerical dispersion, which tends to smear out sharp fronts. Special techniques need to be adapted to alleviate this problem, including using higher-order approximation. For example, Pianet and Arquis [40] used explicit interface reconstruction. Hu et al. [43], on the other hand, introduced an Arbitrary Lagrangian–Eulerian (ALE) technique to track the interface. In this technique, the particle and the fluid are dealt with as separate entities (the realm of the discrete element method).

## 4. The Numerical Scheme

**Figure 4:**
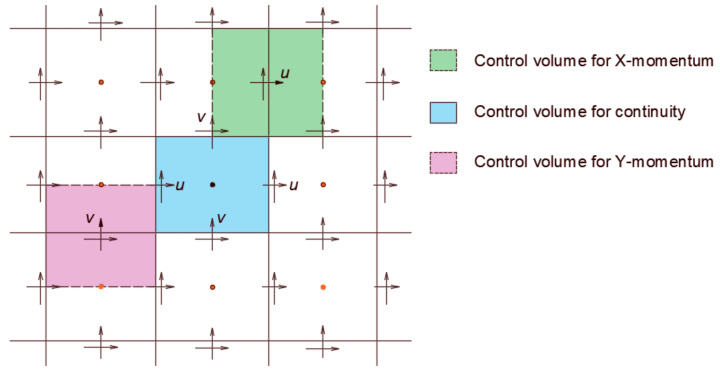
Schematic of the staggered grid for the three equations.

As introduced earlier, the modeling methodology of this problem rests on the one-domain model, in which both the fluid and the solid disk are assumed to be fluids. This roll out the need to consider the boundary condition at the solid–fluid interface and means that the computational grid must include the solid disk. The velocity of the solid domain is, apparently, constrained by the required rigid body motion. The governing equations describing this system, including the continuity and the momentum balance equations (Equations (3) and (6)), are discretized over a staggered grid as shown in Figure 4 [44,45,46,47,48,49]. Several schemes are available for the numerical solution of the N-S equations; see, for example, Gylys et al. [50] and Horwitz and Mani [51]. To numerically solve this system subject to certain boundary conditions, a semi-implicit scheme is developed, such that:

(25) $$\left(1+\Delta tv^{\ell }\cdot \nabla -\Delta tv^{\ell }\nabla^{2}\right)v^{\ell +1}+\frac{\Delta t}{\rho }\nabla p^{\ell +1}=v^{\ell },\;$$

(26) $$\nabla \cdot v^{\ell +1}=0,\;$$

The solution of this system requires a pair of compatible pressure and velocity spaces and the global linear system may be constructed in the form:

(27) $$\left[\begin{array}{cc} A & B\\ B^{T} & 0 \end{array}\right]\left[\begin{array}{c} v\\ p \end{array}\right]=\left[\begin{array}{c} f\\ 0 \end{array}\right].$$

This system, in some cases, can be singular and requires some stabilizing techniques to ensure the invertability of the global matrix. One such technique is to introduce a stabilization matrix, ϵD, and a perturbation to the right-hand side, ϵd—e.g., Salama et al. [47]. In this case, the global system takes the form:

(28) $$\left[\begin{array}{cc} A & B\\ B^{T} & -\epsilon D \end{array}\right]\left[\begin{array}{c} v\\ p \end{array}\right]=\left[\begin{array}{c} f\\ -\epsilon d \end{array}\right].$$

The stabilizing matrix, as shown in the above equation, involves adding a pressure term to the continuity equation. One such possibility has been to consider solving the following continuity equation:

(29) $$\epsilon \frac{\partial p}{\partial t}+\nabla \cdot v=0,\;$$

where ϵ represents a parameter that mimics the compressibility of the fluid.

More details about this technique can be found in Salama et al. [46] and Sun et al. [51]. In this work, we considered the system described by Equation (27); however, in all simulated cases ϵ has been considered zero. The algorithm adapted in this work utilizes the newly developed technique—namely, the experimenting field approach, as in Salama et al. [52]. In this approach, the matrices given in Equation (27) are constructed automatically within the code rather than determined by the user a priori and then emplaced into the global system.

**Figure 5:**
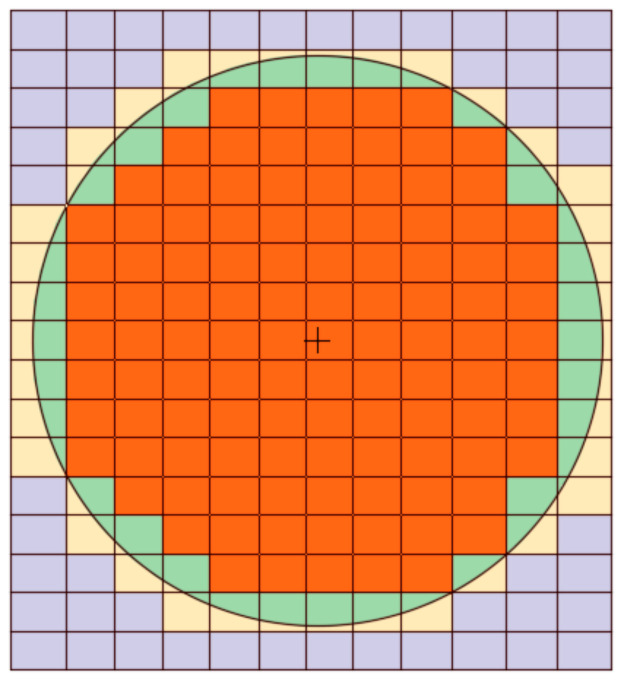
Discrete determination of the average velocity over the cells that are completely inside the boundary.

**Figure 6:**
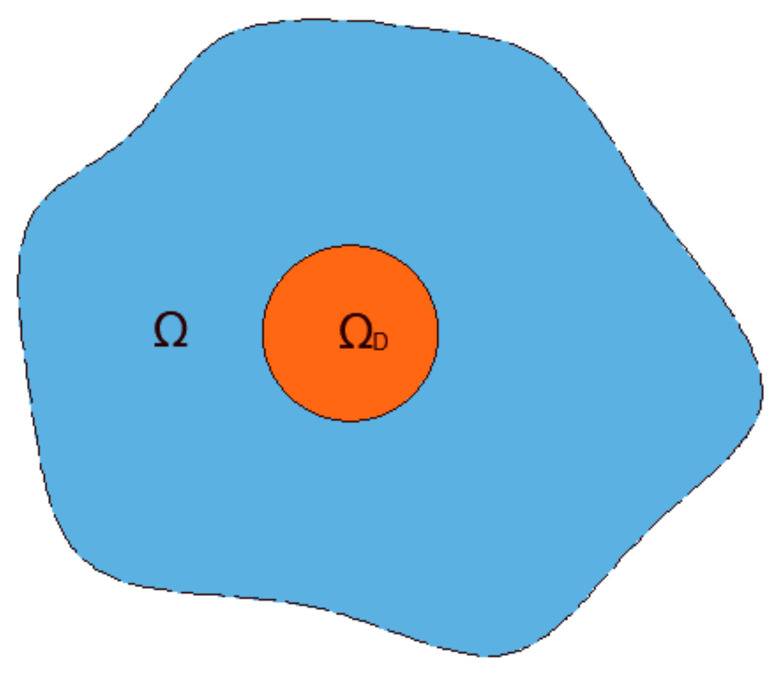
Schematic representation of the domain.

In our case, three fields are designed for the three unknowns—namely, the pressure and the velocities in both x and y directions. These fields have particular character in that they are 1 in the cell of interest (or on the face) and zeros in all other cells (or faces). Therefore, for our 2D case with *n* × *m* cells there are (*nm*) fields for the pressure, (*n* + 1) × *m* for the *u* velocity component, and *n* × (*m* + 1) for the velocity component *v*. More details about this technique can be found in the works of Salama et al. [52]. The velocity of the centroid of the disk is considered the average of the velocity of all the cells contained inside its circular boundary; see Figure 5. They are calculated as:

(30) $${\left.u\right|}_{avg}=\frac{1}{\Omega_{D}}\underset{\Omega_{D}}{\int }ud\Omega ,\;$$

(31) $${\left.v\right|}_{avg}=\frac{1}{\Omega_{D}}\underset{\Omega_{D}}{\int }vd\Omega ,$$

where $\Omega_{D}$ is the domain defining the droplet; see Figure 6. The average velocity is then used to update the position of the particle and to reconstruct the sphere in the new position.

**Figure 7:**
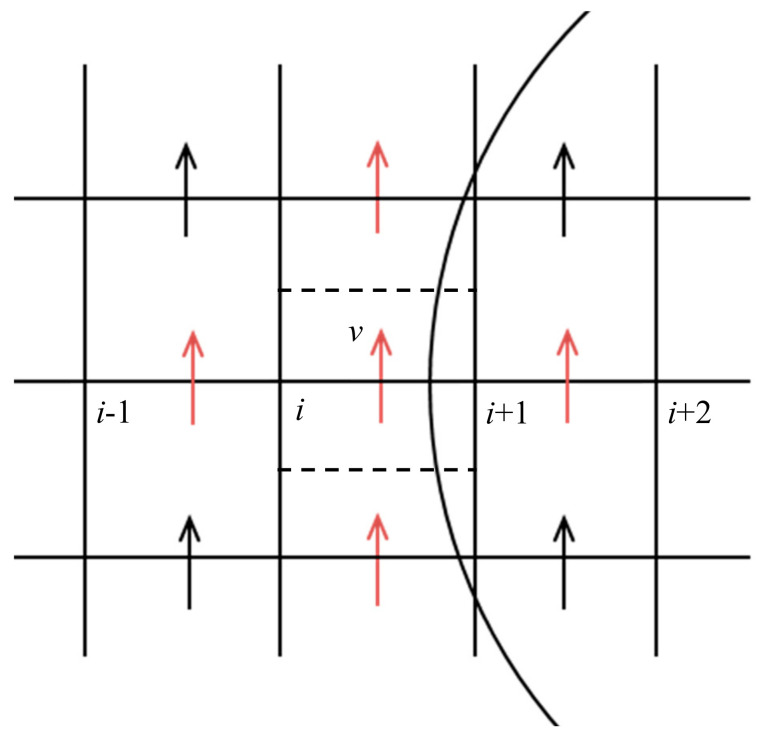
Evaluation of the second order derivative closer to the interface.

As pointed out earlier, the solid particle is treated in this system as a fluid with a density the same as that of the solid and the viscosity orders of magnitudes larger than typical fluid viscosity. This introduces difficulties to the numerical scheme for evaluating properties at cell faces, where a large contrast in the properties exists in adjacent cells. Past experiences with respect to flow in porous media in which permeability field is usually rough over orders of magnitudes reveals that the weighted harmonic mean of permeability is the best choice rather than any other means of averaging (e.g., arithmetic mean). In fact, within the framework of mixed finite element, a harmonic average appears naturally based on some mathematical manipulations [53]. For the sake of comparison, however, we consider both averaging techniques. Consider evaluating the term $\frac{\partial }{\partial x}\mu \frac{\partial v}{\partial x}$ in the neighborhood of the interface (Figure 7); the following expression may be used:

(32) $${\left(\frac{\partial }{\partial x}\mu \frac{\partial v}{\partial x}\right)}_{i+\frac{1}{2},j}=\frac{{\left(\mu \frac{\partial v}{\partial x}\right)}_{i+1}-{\left(\mu \frac{\partial v}{\partial x}\right)}_{i}}{x_{i+1}-x_{i}}=\frac{\mu_{i+1}\left(\frac{v_{i+\frac{3}{2}}-v_{i+\frac{1}{2}}}{x_{i+\frac{3}{2}}-x_{i+\frac{1}{2}}}\right)-\mu_{i}\left(\frac{v_{i+\frac{1}{2}}-v_{i-\frac{1}{2}}}{x_{i+\frac{1}{2}}-x_{i-\frac{1}{2}}}\right)}{x_{i+1}-x_{i}}.\;$$

The viscosity (e.g.,$\;\mu_{i+1}$) at the faces (edges in 2D) is evaluated as:

Weighted harmonic mean:

(33) $$\mu_{i+1}=\frac{x_{i+\frac{3}{2}}-x_{i+\frac{1}{2}}}{\frac{x_{i+1}-x_{i+\frac{1}{2}}}{\mu_{i+\frac{1}{2}}}+\frac{x_{i+\frac{3}{2}}-x_{i+1}}{\mu_{i+\frac{3}{2}}}}$$

Weighted arithmetic mean:

(34) $$\mu_{i+1}=\;w_{i+\frac{1}{2}}\;\mu_{i+\frac{1}{2}}+w_{i+\frac{3}{2}}\;\mu_{i+\frac{3}{2}.}\;$$

where:

(35) $$w_{i+\frac{1}{2}}=\frac{x_{i+1}-x_{i+\frac{1}{2}}}{x_{i+\frac{3}{2}}-x_{i+\frac{1}{2}}},\;$$

(36) $$w_{i+\frac{3}{2}}=\frac{x_{i+\frac{3}{2}}-x_{i+1}}{x_{i+\frac{3}{2}}-x_{i+\frac{1}{2}}}.\;$$

It should be pointed out that other representations of the harmonic mean and weighted arithmetic mean can also be used. The different methods for evaluating the parameters at the cell faces will produce almost the same results in the bulk phases. However, at the interface it can be quite different. The accurate construction of the interface is therefore important. In this work, because the solid object is simply a circle, it suffices to track the center point and construct the object based on the distance to the center.

**Figure 8:**
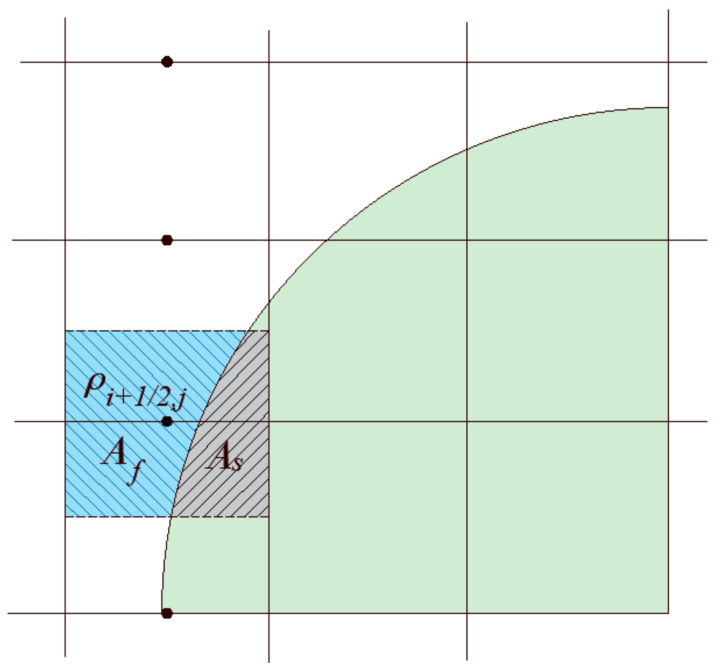
Evaluating the density in the interface region.

For the density evaluation at the edges, we compare two methods; in the first one, a density indicator function is used in which the location at the center of the faces is determined whether it lies in the fluid or in the solid regions, and it is assigned the value of the phase which it lies in at the given time. In the second method, a volume weighted average is used to assign the density at the face centers. This approach requires calculating the volume (area in 2D) partition between phases. As shown in Figure 8, the density at the mid edge is calculated as:

Density indicator function:

(37) $$\rho_{i+\frac{1}{2},j}={\bar{\rho }}_{f}.\;$$

Weighted arithmetic volume average:

(38) $$\rho_{i+\frac{1}{2},j}=w_{f}{\bar{\rho }}_{f}+w_{s}{\bar{\rho }}_{s},\;$$

(39) $$w_{f}=\frac{A_{f}}{A_{cell}},\;$$

(40) $$w_{s}=\frac{A_{s}}{A_{cell}}.\;$$

## 5. Benchmarking and Comparisons

**Figure 9:**
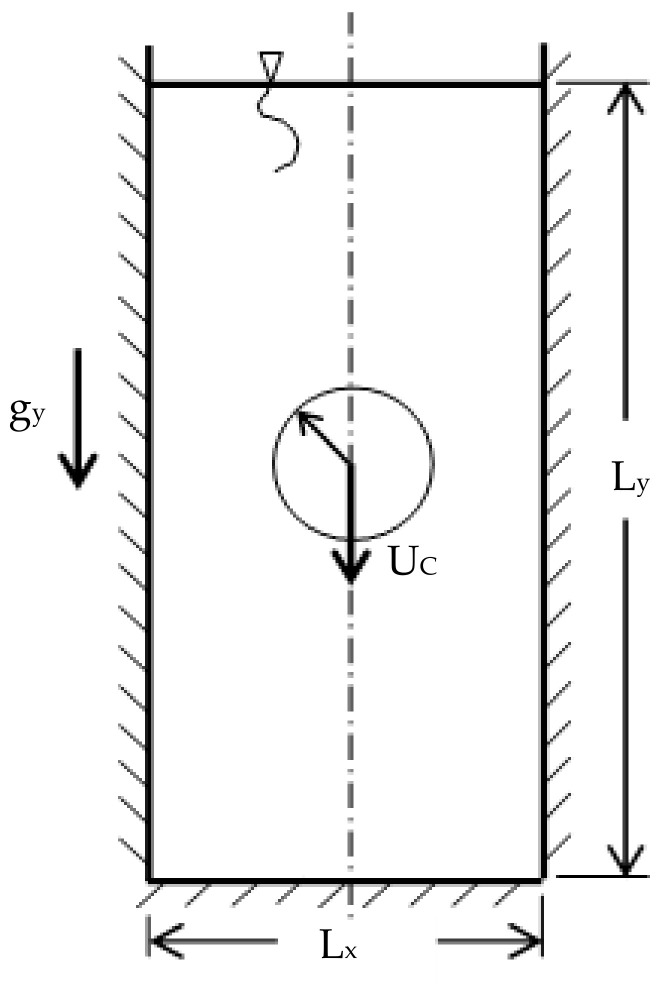
Schematic of the computational domain.

Now, we benchmark the results of this work and compare them with the analytical solution and other simulation results. We show the effects of the different methods to handle the cell face- and cell center-related coefficients on the terminal velocity. The domain of interest is shown in Figure 9 below, which defines a two-dimensional rectangular domain of height Ly and width length L_x_. In the middle of this domain, an extended cylinder of radius *a* is initially inserted and is released to settle under the effect of gravity. The object will initially accelerate until reaching a terminal velocity, which will be maintained for the rest of the motion until the disc encounters the bottom boundary. The contours of some hydrodynamic parameters, such as the vorticity as well as the y-direction velocity profiles at different sections, will be generated.

One of the important geometrical parameters that characterize this system is the ratio of the circle diameter to the width of the rectangular domain—i.e., $k_{x}=d/L_{x}$. This ratio determines the influence of the two confining walls on the terminal velocity of the solid object. The smaller this ratio is the smaller the effect of the walls and vice versa. In this work, we consider $k_{x}$ ratios up to 1:20 to compare with the work of Faxén and Pianet and Arquis [40]. Furthermore, different numerical schemes for estimating the parameters closer to the solid–fluid interface have been tested and the results are reported in the Appendix A. Table 1 below shows a list of the considered options for calculating the parameters (density and viscosity) at the face centers. Moreover, the sensitivity of the terminal velocity calculations to the mesh resolution is also considered and reported in the Appendix A, as well as the effect of the value chosen for the viscosity of the solid disk.

For convenience, we consider the case when a solid particle of diameter 0.05 m is released from rest with initial velocity zero into a quiescent fluid; the parameters used in this simulation are given in Table 2. In another example, the diameter of the 2D extended cylinder is reduced to 0.02 m, fixing the other parameters. Apparently, the size here represents upscaled particle and domain sizes. For typical particle sizes in produced water applications, a downscaling of both the particle and the domain sizes would be required, but the conclusion would still be the same. It is to be noted that, for the case of droplets, no analytical solution, such as that developed by Faxen for a particle in a confined space, exists. Some data of large viscosity contrast that are not infinite can be seen in the Appendix A. In this case, the droplet very much resembles that of a solid particle. The case of smaller viscosity contrast is the work of future research. The conclusion is, therefore, valid for both solid particles and droplets.

**Figure 10:**
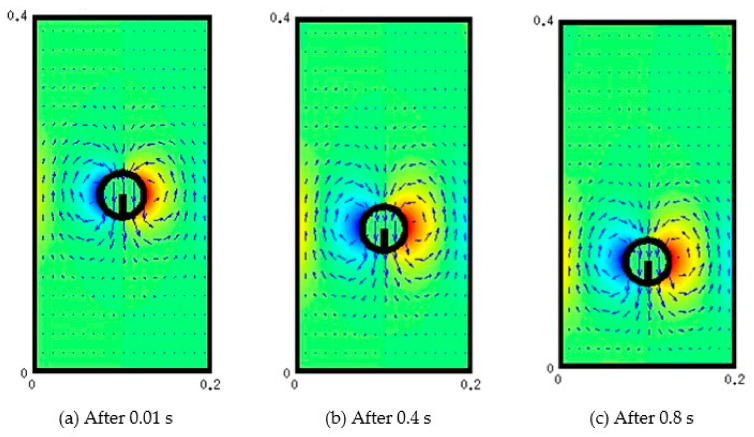
Succession of particle motion (*ρ_s_* = 3000, *ρ_f_* = 1000, μ_s_ = 1000, μ_f_ = 17.72).

**Figure 11:**
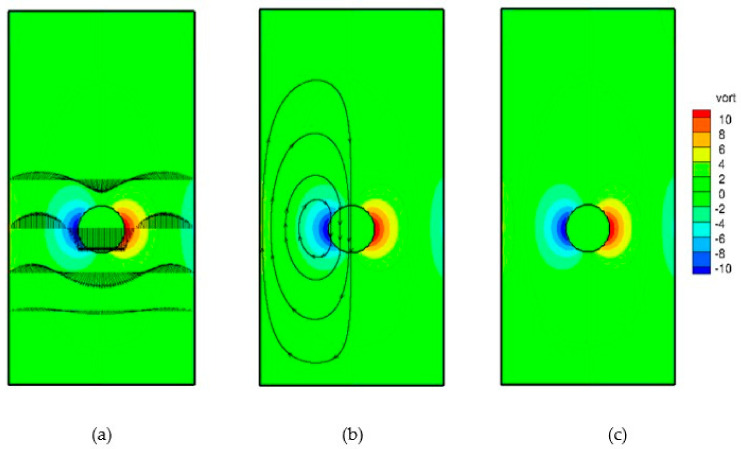
Velocity profiles (**a**), streamlines (**b**), and vorticity contours (**c**).

The results of benchmarking along with the accuracy and comparisons of the different techniques to calculate properties at the interface are documented in Appendix A. Figure 10 below shows a succession of snapshots of the particle position and velocity field associated with the motion of the particle. Figure 11a shows the patterns of velocity profiles along the width of the channel. The velocity field in the central part is downward and is upward towards the vertical walls. This result in two recirculation zones in each half of the channel, as depicted in Figure 11b.

Another important measure of the flow field is related to the amount of rotation of fluid elements and the location where the rotation is large. Such tendency for fluid elements to spin defines an important hydrodynamic quantity, which is called the vorticity. It can be related to the local angular rate of rotation in a fluid and is defined as the curl of the velocity field, or:

(41) ω=∇×v.

Its components are defined as:

(42) $$\omega =\left(\frac{\partial w}{\partial y}-\frac{\partial v}{\partial z},\frac{\partial u}{\partial z}-\frac{\partial w}{\partial y},\frac{\partial v}{\partial x}-\frac{\partial u}{\partial y}\right).\;$$

In two dimensions, only one component exists normal to the plane of motion—i.e.,:

(43) $$\omega =\left(0,0,\frac{\partial v}{\partial x}-\frac{\partial u}{\partial y}\right)$$

This component of vorticity defines the circulation about a closed contour in the horizontal plane divided by the area enclosed; in the limit where the area approaches zero, we have:

(44) $$\xi =\underset{\Delta A\to 0}{\lim}\frac{\oint v\cdot dl}{\Delta A}.\;$$

**Figure 12:**
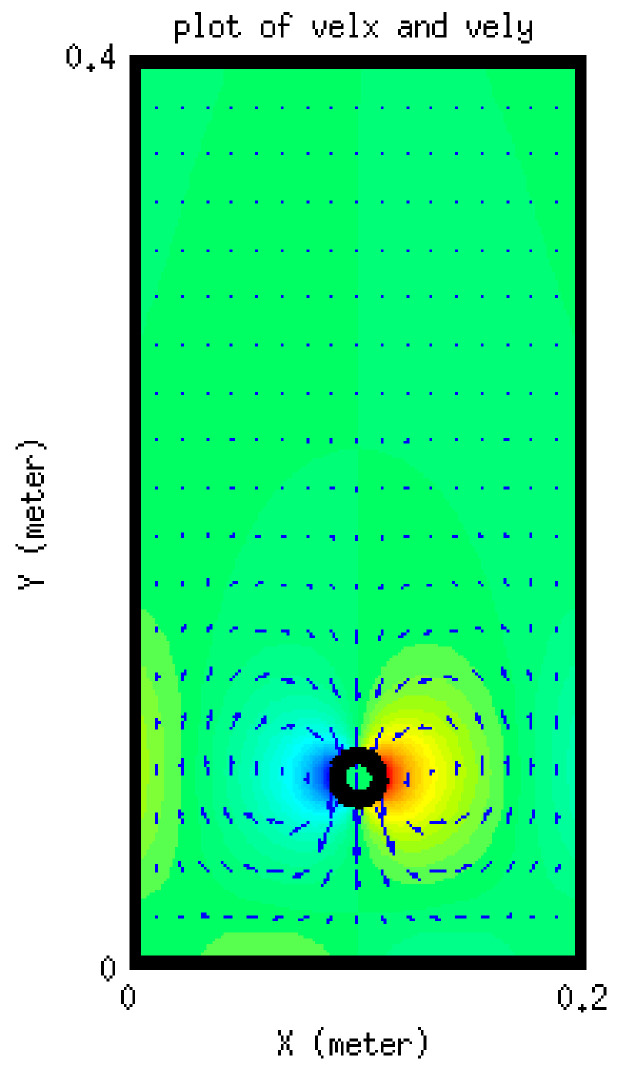
Velocity vectors of the fluid.

**Figure 13:**
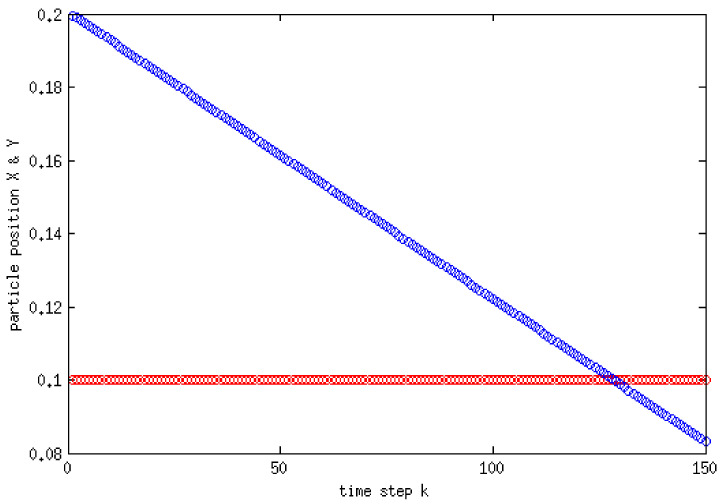
x (red) and y (blue) positions of the particle with time.

**Figure 14:**
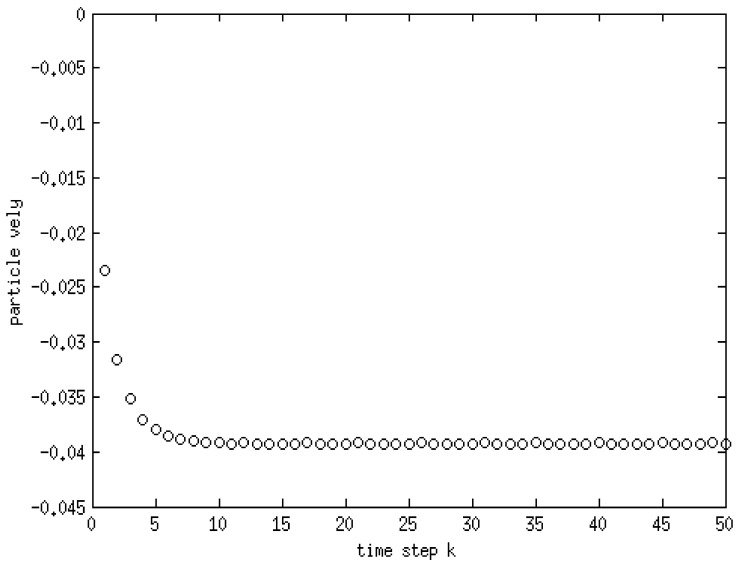
Y-component velocity with time.

Vorticity contours are shown in Figure 11c, from which it is clear that the area in the vicinity of the settling object encounters the largest circulation. The velocity vectors of the fluid are also shown Figure 12. It can be seen that the velocity vectors are relatively large in the vicinity of the particle compared with that far from the particle. The profile of the components of the position of the particle with time is depicted in Figure 13. This shows the location of the centroid of the solid particle when the diameter is reduced to 0.02 m after 0.4 s. It is clear that the particle stays at the center line (constant x-coordinate) while the y-coordinate decreases with time. Figure 14, on the other hand, shows the y-component velocity magnitude, from which it is clear that the velocity reaches a terminal value as the time proceeds.

**Figure 15:**
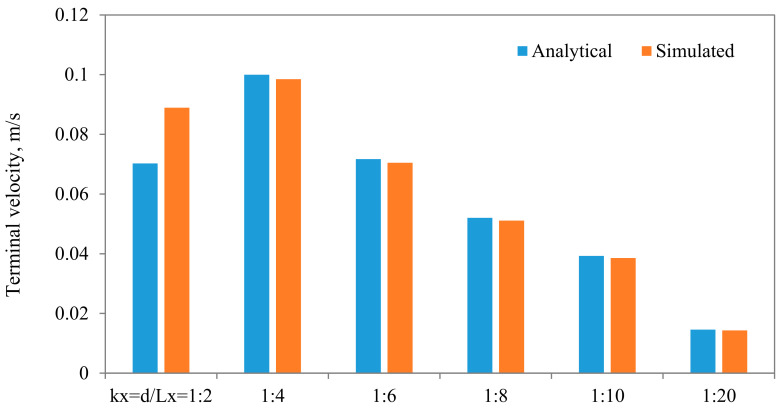
Comparisons between the terminal settling velocity and the simulated one for different diameter to width ratios. In these simulations, the following parameters are held fixed: μ_f_ = 0.001 Pa·s, μ_p_ = 1000 Pa·s, Mesh n_x_ = 40, n_y_ = 80.

**Figure 16:**
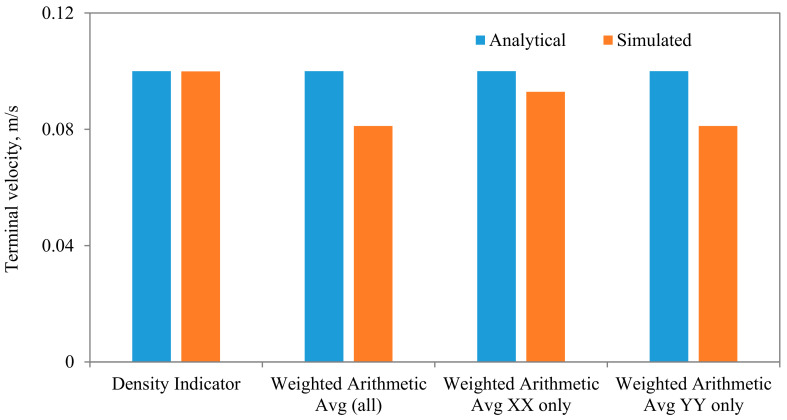
Comparisons between the terminal settling velocity and the simulated one for different algorithms for calculating the density in the cells encompassing the interface. In these simulations, the following parameters are held fixed: $k_{x}$ = d/L_x_ = 4, μ_f_ = 0.001 Pa·s, μ_p_ = 1000 Pa·s, Mesh n_x_ = 40, n_y_ = 80.

**Figure 17:**
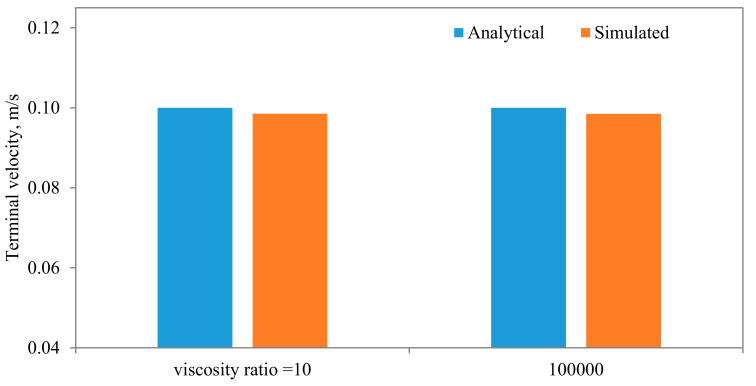
Comparisons between the terminal settling velocity and the simulated one for different viscosity ratios. In these simulations, the following parameters are held fixed: $k_{x}$ = d/L_x_ = 4, μ_f_ = 0.001 Pa·s, Mesh density n_x_ = 40, n_y_ = 80.

**Figure 18:**
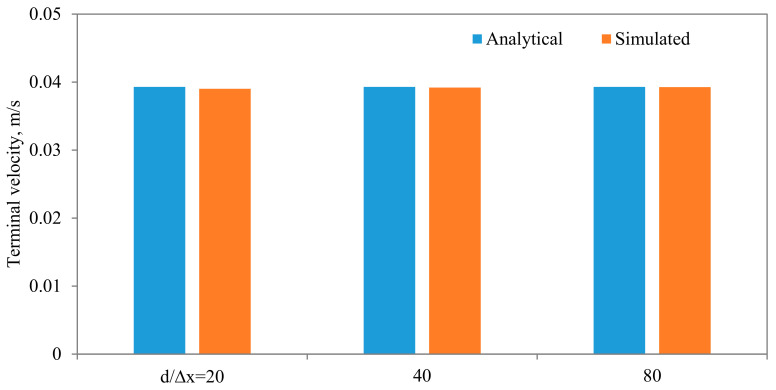
Comparisons between the terminal settling velocity and the simulated one for different spatial resolutions. In these simulations, the following parameters are held fixed: $k_{x}$ = d/L_x_ = 4, μ_f_ = 0.001 Pa·s, μ_p_ = 1000 Pa·s.

Comparisons between the terminal velocities calculated using Equations (22) and (23) and that simulated assuming different diameter to width ratios, for the same parameters and mesh resolution, are shown in Figure 15. As can be seen, very good agreements can be observed, except when the diameter of the particle is closer to the width of the channel. The influences of the algorithm for calculating the density of the fluid in the cell that encompass the interface are shown in Figure 16, from which it is evident that the density calculated using the density indicator is more accurate. This should come as no surprise, as the volume of the cell occupied by one fluid versus the other volume is utilized in the calculation of the density. Figure 17 shows the effect of the viscosity ratio between the two fluids. It is clear that the viscosity contrast does not seem to have a significant effect for the range considered in this work. This is also in conformity of the finding of Salama [16,17,18] upon the study of the critical velocity of dislodgment of the breakup scenario of a permeating velocity. The effects of the spatial resolution of the mesh are depicted in Figure 18, from which it is clear that the developed algorithm is accurate even with a relatively coarse mesh. This is a great advantage, as regular sharp interface models require exhaustively denser meshes to track and reconstruct the interface [10,11,12,13,14]. Furthermore, they confront difficulties when there are large contrasts in properties (e.g., large viscosity contrast between the two fluids). In this approach, such difficulties do not exist and the model can span a large spectrum of contrasts from the solid particle limit to droplets.

## 6. Conclusions

A modeling approach based on the one-domain assumption has been developed to investigate the problem of drag over a two-dimensional fluid object by another outside fluid. In this model, fluid with heterogeneous properties is considered filling up the entire domain. The heterogeneity is abrupt at the boundary between phases and is homogeneous elsewhere. The geometry of the fluid object is assumed to be fixed—i.e., nondeforming—which may be valid under restricted conditions (e.g., smaller capillary number scenario). In this case, the capillary force may not be important and may, therefore, be ignored from the governing equations. This very much simplifies the numerical algorithm and alleviates the need to update the interface according to the evolution of a phase-field function.

In order to build confidence in our modeling approach and the accuracy of the developed numerical scheme, a comprehensive benchmarking of the results obtained from this model and available data from the literature has been conducted. It is clear that viscosity contrast $\lambda =\mu_{i}/\mu_{o}$, where $\mu_{i}$ and $\mu_{o}$ are the viscosities of the inside and outside fluids, respectively, plays a crucial role in the gripping of the outside fluid by the inside one. In the limit when the viscosity contrast approaches infinity, this represents the rigid object scenario and the inside fluid behaves as a solid object. The viscosity contrast, in other words, has been used as a parameter whose extreme values represent either a solid or a gas phase. The problem, however, has been in handling these quantities at the interface. Traditional sharp interface models (e.g., VOF method) face difficulties when the viscosity contrast is large, which has not been observed when using this approach.

To provide benchmarking with the available literature and to build confidence in the numerical approach, ample numerical experiments have been conducted. The case of moving a semi-infinite cylindrical object in a confined, initially quiescent fluid which has an analytical solution has been used to benchmark this approach. Comparisons have been conducted between this approach and the analytical solutions, and very good match was obtained. Several scenarios to evaluate the parameters at the nodes closer to the solid wall were tested.

## Figures and Tables

**Table 1:** List of the considered averaging options for density and viscosity.

	μ	ρ
Harmonic average	x	
Weighted arithmetic average, only $\left(\tau_{\mathrm{xx}},{\;\tau }_{\mathrm{yy}}\right)$	x	
Weighted arithmetic average, only $\left(\tau_{\mathrm{xy}},{\;\tau }_{\mathrm{yx}}\right)$	x	
Weighted arithmetic average, all ($\tau_{\mathrm{xx}},{\;\tau }_{\mathrm{yy}},\tau_{\mathrm{xy}},{\;\tau }_{\mathrm{yx}}$)	x	
Weighted volume average		x
Density indicator		x

**Table 2:** Parameters used in the first modeled scenario.

L_x_ (m)	L_y_ (m)	d (m)	$k_{x}$ = d/L_x_	*ρ*_s_	*ρ*_f_	μ_f_ (Pa.s)	μ_s_ (Pa.s)	Mesh (nx,ny)
0.2	0.4	0.05	1:4	3000	1000	17.52	10^3^	200,400

## Data Availability

Data is contained within the article.

## Appendix A. (Results of Benchmarking)

**Parameters:** Radius = a, Diameter = d, Width = L_x_, Height= L_y_. The colored cells represent the variables investigated.

**Table A1:** Base Scenario.

L_x_ (m)	L_y_ (m)	$k_{x}=d/L_{x}$	d/∆x	μ_f_ (Pa.s)	μ_s_ (Pa.s)	Mesh (nx,ny)
0.2	0.4	1:4	10	10^−3^	10^3^	40,80

**Table A2:** Effect of the ratio *k* = d/L_x_.

Case	U_c_, Analytical (m/s)	U_p_, Simulated (m/s)	% Relative Error
$k_{x}$ = d/L_x_ = 1:2	−0.0702567	−0.0889332	26.5833
1:4	−0.0999891	−0.0984975	1.49169
1:6	−0.071729	−0.0704685	1.75721
1:8	−0.0520517	−0.0511041	1.82054
1:10	−0.0392939	−0.38567	1.85011
1:20	−0.0145841	−0.014307	1.89984

**Table A3:** Numerical Algorithm for interface cells.

**L_x_ (m)**	**L_y_ (m)**	**$k_{x}$ = d/L_x_**	**d/∆x**	**μ_f_ (Pa.s)**	**μ_s_ (Pa.s)**	**Mesh (nx,ny)**
0.2	0.4	1:4	10	10^−3^	10^3^	40,80
Case		U_c_, Analytical (m/s)		U_p_, Simulated (m/s)	% relative error	
With density Indicator		−0.0999891		−0.0999057	0.0833489	
Weighted Arithmetic Avg (all)		−0.0999891		−0.0811345	18.8566	
Weighted Arithmetic Avg XX only		−0.0999891		−0.0929154	7.0744	
Weighted Arithmetic Avg XY only		−0.0999891		−0.0811345	18.8566	
**L_x_ (m)**	**L_y_ (m)**	**$k_{x}$ = d/L_x_**	**d/∆x**	**μ_f_ (Pa.s)**	**μ_s_ (Pa.s)**	**Mesh (nx,ny)**
0.2	0.4	1:6	10	10^−3^	10^3^	40,80
Case		U_c_, Analytical (m/s)		U_p_, Simulated (m/s)	% relative error	
With density Indicator		−0.071729		−0.0714543	0.382922	
Weighted Arithmetic Avg		−0.071729		−0.0617425	13.9225	
Weighted Arithmetic Avg XX only		0.071729		−0.0675415	5.8379	
Weighted Arithmetic Avg XY only		−0.0999891		−0.0617425	13.922	

**Table A4:** Effect of Viscosity.

**Case I**						
**L_x_ (m)**	**L_y_ (m)**	**$k_{x}$ = d/L_x_**	**d/∆x**	**μ_f_ (Pa.s)**	**μ_s_ (Pa.s)**	**Mesh (nx,ny)**
0.2	0.4	1:4	10	10^−3^	10^5^	40,80
Case		U_c_, Analytical (m/s)		U_p_, Simulated (m/s)	% relative error	
With density Indicator		−0.0999891		−0.0984977	1.49151	
**Case II**						
**L_x_ (m)**	**L_y_ (m)**	**$k_{x}$ = d/L_x_**	**d/∆x**	**μ_f_ (Pa.s)**	**μ_s_ (Pa.s)**	**Mesh (nx,ny)**
0.2	0.4	1:4	10	10^−3^	10	40,80
Case		U_c_, Analytical (m/s)		U_p_, Simulated (m/s)	% relative error	
With density Indicator		−0.0999891		−0.0985065	1.49151	

**Table A5:** Effect of mesh resolution.

**L_x_ (m)**	**L_y_ (m)**	**$k_{x}$ = d/L_x_**	**d/∆x**	**μ_f_ (Pa.s)**	**μ_s_ (Pa.s)**
0.2	0.4	1:2	20	10^−3^	10^5^
Case	U_c_, Analytical (m/s)		U_p_, Simulated (m/s)	% relative error	
With density Indicator	−0.0702567		−0.088169	25.4956	
**L_x_ (m)**	**L_y_ (m)**	**$k_{x}$ = d/L_x_**	**d/∆x**	**μ_f_ (Pa.s)**	**μ_s_ (Pa.s)**
0.2	0.4	1:2	40	10^−3^	10^5^
Case	U_c_, Analytical (m/s)		U_p_, Simulated (m/s)	% relative error	
With density Indicator	−0.0702567		−0.0883669	25.7772	
**L_x_ (m)**	**L_y_ (m)**	**$k_{x}$ = d/L_x_**	**d/∆x**	**μ_f_ (Pa.s)**	**μ_s_ (Pa.s)**
0.2	0.4	1:2	80	10^−3^	10^5^
Case	U_c_, Analytical (m/s)		U_p_, Simulated (m/s)	% relative error	
With density Indicator	−0.0702567		−0.0883825	25.7994	
**L_x_ (m)**	**L_y_ (m)**	**$k_{x}$ = d/L_x_**	**d/∆x**	**μ_f_ (Pa.s)**	**μ_s_ (Pa.s)**
0.2	0.4	1:2	160	10^−3^	10^5^
Case	U_c_, Analytical (m/s)		U_p_, Simulated (m/s)	% relative error	
With density Indicator	−0.0702567		−0.0884388	25.8796	
**L_x_ (m)**	**L_y_ (m)**	**$k_{x}$ = d/L_x_**	**d/∆x**	**μ_f_ (Pa.s)**	**μ_s_ (Pa.s)**
0.2	0.4	1:6	20	10^−3^	10^5^
Case	U_c_, Analytical (m/s)		U_p_, Simulated (m/s)	% relative error	
With density Indicator	−0.071729		−0.0712175	0.712979	
**L_x_ (m)**	**L_y_ (m)**	**$k_{x}$ = d/L_x_**	**d/∆x**	**μ_f_ (Pa.s)**	**μ_s_ (Pa.s)**
0.2	0.4	1:6	40	10^−3^	10^5^
Case	U_c_, Analytical (m/s)		U_p_, Simulated (m/s)	% relative error	
With density Indicator	−0.071729		−0.0715578	0.23855	
**L_x_ (m)**	**L_y_ (m)**	**$k_{x}$ = d/L_x_**	**d/∆x**	**μ_f_ (Pa.s)**	**μ_s_ (Pa.s)**
0.2	0.4	1:6	80	10^−3^	10^5^
Case	U_c_, Analytical (m/s)		U_p_, Simulated (m/s)	% relative error	
With density Indicator	−0.071729		−0.0716596	0.0967284	
**L_x_ (m)**	**L_y_ (m)**	**$k_{x}$ = d/L_x_**	**d/∆x**	**μ_f_ (Pa.s)**	**μ_s_ (Pa.s)**
0.2	0.4	1:10	20	10^−3^	10^5^
Case	U_c_, Analytical (m/s)		U_p_, Simulated (m/s)	% relative error	
With density Indicator	−0.0392939		−0.0390103	0.721781	
**L_x_ (m)**	**L_y_ (m)**	**$k_{x}$ = d/L_x_**	**d/∆x**	**μ_f_ (Pa.s)**	**μ_s_ (Pa.s)**
0.2	0.4	1:10	40	10^−3^	10^5^
Case	U_c_, Analytical (m/s)		U_p_, Simulated (m/s)	% relative error	
With density Indicator	−0.0392939		−0.0391964	0.248302	
**L_x_ (m)**	**L_y_ (m)**	**$k_{x}$ = d/L_x_**	**d/∆x**	**μ_f_ (Pa.s)**	**μ_s_ (Pa.s)**
0.2	0.4	1:10	80	10^−3^	10^5^
Case	U_c_, Analytical (m/s)		U_p_, Simulated (m/s)	% relative error	
With density Indicator	−0.0392939		−0.0392554	0.0981869	
